# Alcohol flushing syndrome is significantly associated with intracranial aneurysm rupture in the Chinese Han population

**DOI:** 10.3389/fneur.2023.1118980

**Published:** 2023-03-17

**Authors:** Xiheng Chen, Siming Gui, Dingwei Deng, Linggen Dong, Longhui Zhang, Dachao Wei, Jia Jiang, Huijian Ge, Peng Liu, Ming Lv, Youxiang Li

**Affiliations:** ^1^Beijing Neurosurgical Institute, Capital Medical University, Beijing, China; ^2^Department of Neurosurgery, Beijing Tian Tan Hospital, Capital Medical University, Beijing, China; ^3^Beijing Engineering Research Center for Interventional Neuroradiology, Beijing, China

**Keywords:** alcohol flushing syndrome, intracranial aneurysm, rupture, risk factor, Chinese Han population

## Abstract

**Objective:**

Although alcohol flushing syndrome (AFS) has been associated with various diseases, its association with intracranial aneurysm rupture (IAR) is unclear. We aimed to examine this association in the Chinese Han population.

**Methods:**

We retrospectively reviewed Chinese Han patients with intracranial aneurysms who were evaluated and treated at our institution between January 2020 and December 2021. AFS was determined using a semi-structured telephone interview. Clinical data and aneurysm characteristics were assessed. Univariate and multivariate logistic regression were conducted to determine independent factors associated with aneurysmal rupture.

**Results:**

A total of 1,170 patients with 1,059 unruptured and 236 ruptured aneurysms were included. The incidence of aneurysm rupture was significantly higher in patients without AFS (*p* < 0.001). Meanwhile, there was a significantly difference between the AFS and non-AFS group in habitual alcohol consumption (10.5 vs. 27.2%, *p* < 0.001). In the univariate analyses, AFS [odds ratio (OR) 0.49; 95% confidence interval (CI), 0.34–0.72] was significantly associated with IAR. In the multivariate analysis, AFS was an independent predictor of IAR (OR 0.50; 95%, CI, 0.35–0.71). Multivariate analysis revealed that AFS was an independent predictor of IAR in both habitual (OR 0.11; 95% CI, 0.03–0.45) and non-habitual drinkers (OR 0.69; 95% CI, 0.49–0.96).

**Conclusion:**

Alcohol flushing syndrome may be a novel clinical marker to assess the risk of IAR. The association between AFS and IAR exists independently of alcohol consumption. Further single nucleotide polymorphism testing and molecular biology studies are warranted.

## Introduction

The estimated prevalence of unruptured intracranial aneurysms (UIAs) ranges between 3 and 7% (1–3) and approximately 1–2% of these aneurysms will progress and rupture (4, 5). Mortality of rupture approaches 40% (6). Detection of UIAs has increased with the widespread use of magnetic resonance angiography (7). Although open surgical or endovascular treatment can prevent rupture, each approach is associated with potential complications, including death and permanent disability. Early identification of rupture risk factors and treatment before rupture are critical. Age, sex, smoking, hypertension, history of previous rupture, and aneurysm size, shape, and location are known factors associated with rupture risk (8–10). However, the mechanisms impacting progression and rupture are complex and multidimensional and there are no effective methods to guide clinical decision making when managing UIAs. Identification of clinical indicators that affect UIA stability would be beneficial.

Yang et al. (11) recently reported in two independent case–control studies that the prevalence of aortic aneurysm/dissection was significantly lower in individuals with mutations in the mitochondrial aldehyde dehydrogenase 2 (ALDH2) gene than in those without mutations, which provides very valuable insight into the study of UIAs. The ALDH2*2 mutation, which substitutes glutamate for lysine at position 504 (Glu504Lys), is unique to East Asians and is present in up to 40% of the East Asian population and almost absent in other populations. East Asian individuals with this mutation develop facial flushing, headache, palpitations, and nausea after drinking alcohol, a reaction known as alcohol (ethanol) flushing syndrome (AFS) (12–17). Presence of AFS can identify ALDH2 deficiency in East Asians and be used as an alternative marker for ALDH2 gene variants (16–18). Numerous studies have shown that ALDH2 polymorphisms are associated with a wide range of neurodegenerative, cerebrovascular, and cardiovascular diseases (13, 19–22). However, the association of AFS and UIA rupture risk is unclear. This study aimed to examine this potential association in Chinese Han patients with intracranial aneurysms.

## Materials and methods

### Subjects

**Figure 1:**
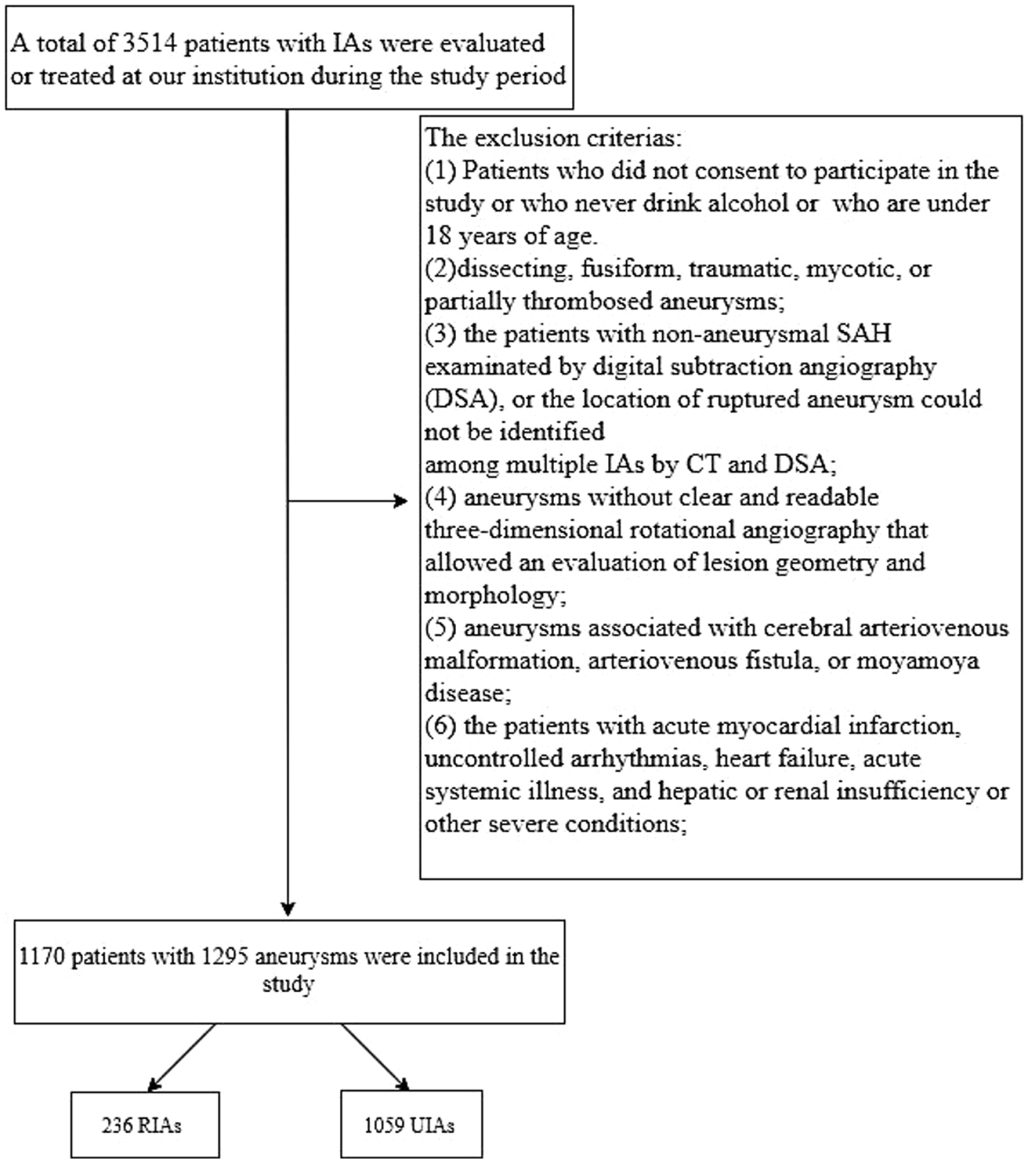
Study flowchart. IA, intracranial aneurysm; SAH, subarachnoid hemorrhage; CT, computed tomography; DSA, digital subtraction angiography; RIA, ruptured intracranial aneurysm; UIA, unruptured intracranial aneurysm.

We retrospectively reviewed all Chinese Han patients with intracranial aneurysms who were evaluated or treated at Beijing Tiantan Hospital between January 2020 and December 2021. All aneurysms were diagnosed using digital subtraction angiography (DSA). Intracranial aneurysm rupture (IAR) was diagnosed based on computed tomography (CT) evidence of subarachnoid hemorrhage. Institutional review board approval was obtained. Among the 3,514 patients who were evaluated or treated, 1,170 patients harboring 1,059 UIAs and 236 ruptured intracranial aneurysms (RIAs) were included for analysis after applying our exclusion criteria. Figure 1 shows the study flowchart and exclusion criteria in detail.

### Data collection

A semi-structured telephone interview (Table 1) was performed by trained interviewers to determine smoking habits, drinking habits, presence of facial flushing when drinking (23–25). Medical history data was collected from treating physicians through interviews with patients and/or family members. Participants were defined as ever-drinkers if they had ever consumed an alcoholic beverage during their lifetime and as never-drinkers if they had never done so. Ever-drinkers were asked about AFS using the following question: ‘Do you have a propensity to experience facial flushing immediately after drinking a glass of beer or an equivalent alcoholic beverage?’ If the answer was “yes, “they were categorized as having AFS; those who answered “no” were categorized as not having AFS. Habitual alcohol drinking was defined as drinking alcohol more than 5 days a week (24). Current and past smokers were defined as smokers. Patients were considered to have diabetes if they had a previously measured 2-h blood glucose concentration ≥ 200 mg/dl after oral glucose tolerance testing or if they were using insulin or oral hypoglycemic drugs. Patients were considered hypertensive if they had a history of untreated hypertension, used antihypertensive medication, or had been diagnosed with hypertension by a physician. Patients were considered to have hyperlipidemia if they had a history of hyperlipidemia, used anti-lipidemic medications, or had been diagnosed with hyperlipidemia by a physician. Patients were considered to have heart disease if they had a history of myocardial infarction, angina pectoris, percutaneous transluminal coronary angioplasty, or coronary artery bypass graft surgery (26).

**Table 1:** The AFS questionnaire.

1.Do you smoke?
A. Yes
B. Used to smoke, but have quit
C. Never
2.If you smoke, how many cigarettes do you smoke on average per day?
A. 1–5 cigarettes
B. 6–10 cigarettes
C. 11–20 cigarettes
D. 20 cigarettes or more
3.Do you drink alcohol?
A. Yes
B. Occasionally
C. No
4.If you drink alcohol, do you drink more than 5 days a week?
A. Yes
B. No
5.If you drink alcohol, do you have a propensity to experience facial flushing immediately after drinking a glass of beer or an equivalent alcoholic beverage?
A. Yes
B. No

AFS, alcohol flushing syndrome.

### Definition of parameters and measurement methods

Two experienced neurointerventionalists evaluated the morphological features of aneurysms using three-dimensional rotational digital subtraction angiography (DSA). The aneurysm size, neck size, and parent artery diameter were measured, and aneurysm shape and location were documented. The maximum cross-sectional diameter, vertical distance between the aneurysm dome and neck, and maximum horizontal diameter were defined as aneurysm size, height, and width, respectively. Aspect ratio (AR) was calculated as the ratio of aneurysm height to neck diameter. Height/width ratio (HWR) was calculated as the ratio of aneurysm height to aneurysm width. Size ratio (SR) was calculated as the ratio of aneurysm size to parent artery diameter. Additionally, the mean vessel diameter was determined by averaging the diameters of two vessel segments upstream of the aneurysm (D1 at the proximal neck and D2 at the upstream 1.5 × D1), (27). A bifurcation aneurysm was defined as originating from an arterial junction (28). These morphological parameters were used to characterize the aneurysms and were analyzed according to established conventions. The measurements were performed by the two neurointerventionalists to ensure consistency, and the inter-observer agreement was assessed.

### Statistical analyses

Continuous variables were analyzed using appropriate statistical tests based on their distribution, such as means with standard deviation for normally distributed variables and median with interquartile range for non-normally distributed variables. Categorical variables were presented as numbers with frequency and compared using chi-square or Fisher’s exact test as appropriate. To assess the relationship between clinical and aneurysm characteristics and aneurysmal rupture, univariate and multivariate logistic regression analyses were performed to calculate odds ratios (ORs) with 95% confidence intervals (CIs). SPSS software version 25 (IBM Corp., Armonk, NY, United States) was used for all statistical analyses. Statistical significance was considered at a *p*-value less than 0.05.

## Results

Patient characteristics are shown in Table 2. Mean patient age was 55.22 ± 10.07 years and 59.9% were women. Habitual alcohol drinking was reported by 21.4%. Thirty-five percent of patients were categorized as having AFS.

**Table 2:** Patient characteristics.

Characteristic	Total patients (*n* = 1,170)	Unruptured aneurysms (*n* = 934 patients)	Ruptured aneurysms (*n* = 236 patients)
Age, mean ± SD	55.2 ± 10.1	55.5 ± 10.0	54.2 ± 10.4
< 50, *n* (%)	327(28.0)	253 (27.1)	74 (31.4)
≥ 50, *n* (%)	843 (72.1)	681 (72.9)	162 (68.6)
Sex
Male, *n* (%)	469 (40.1)	352 (37.7)	117 (49.6)
Female, *n* (%)	701 (59.9)	582 (62.3)	119 (50.4)
History of diabetes mellitus, *n* (%)	150 (12.8)	121 (13.0)	29 (12.3)
History of hypertension, *n* (%)	660 (56.4)	518 (55.5)	142 (60.2)
History of hyperlipidemia, *n* (%)	401 (34.3)	331 (35.4)	70 (29.7)
History of heart disease, *n* (%)	135 (11.5)	106 (11.3)	29 (12.3)
History of ischemic stroke, *n* (%)	154 (13.2)	138 (14.8)	16 (6.8)
Smoking, *n* (%)	339 (29.0)	248 (26.6)	91 (38.6)
Alcohol habit, *n* (%)	250 (21.4)	178 (19.1)	72 (30.5)
AFS, *n* (%)	410 (35.0)	354 (37.9)	56 (23.7)
Multiplicity, *n* (%)	125 (10.7)	92 (9.9)	33 (14.0)

AFS, alcohol flushing syndrome; SD, standard deviation.

Aneurysm characteristics overall and according to rupture status are summarized in Table 3. Overall, 90.4% were located in the anterior circulation. Among ruptured aneurysms, 86.9% were located at a bifurcation, 52.5% were greater than 7 mm in diameter, and 71.2% were irregularly shaped.

**Table 3:** Aneurysm characteristics.

Characteristic	Total aneurysms (*n* = 1,295)	Unruptured aneurysms (*n* = 1,059)	Ruptured aneurysms (*n* = 236)
Locations of aneurysms, *n* (%)
Anterior circulation aneurysms, *n* (%)	1,171 (90.4)	969 (91.5)	202 (85.6)
ICA	587 (45.3)	568 (53.6)	19 (8.1)
ACOM	169 (13.1)	104 (9.8)	65 (27.5)
PCOMA	202 (15.6)	125 (11.8)	77 (32.6)
MCA	159 (12.3)	131 (12.4)	28 (11.9)
ACA	54 (4.2)	41 (3.9)	13 (5.5)
Posterior circulation aneurysms^*^, *n* (%)	124 (9.6)	90 (8.5)	34 (14.4)
Bifurcation aneurysm, *n* (%)
No	561 (43.3)	530 (50.0)	31 (13.1)
Yes	734 (56.7)	529 (50.0)	205 (86.9)
Size of largest aneurysm (mm)
< 7 mm, *n* (%)	910 (70.3)	798 (75.4)	112 (47.5)
≥ 7 mm, *n* (%)	385 (29.7)	261 (24.6)	124 (52.5)
Aneurysm shape
Regular shape, *n* (%)	725 (56.0)	657 (62.0)	68 (28.8)
Irregular shape, *n* (%)	570 (44.0)	402 (38.0)	168 (71.2)
AR, median [IQR]	0.99 [0.86,1.18]	0.96 [0.85,1.15]	1.11 [0.93,1.34]
SR, median [IQR]	1.75 [1.16,2.85]	1.56 [1.06,2.44]	2.976 [2.04,4.22]
HWR, median[IQR]	1.39 [1.09,1.80]	1.35 [1.07,1.77]	1.57 [1.22,2.07]

* posterior circulation aneurysms comprised those located at the basilar tip, basilar-superior cerebellar artery junction, vertebral-posterior inferior cerebellar artery junction, and vertebrobasilar junction.

ACA, anterior cerebral artery; ACOM, anterior cerebral-anterior communicating artery junction; AR, aspect ratio; HWR, height-width ratio; IQR, interquartile range; ICA, internal carotid artery; MCA, middle cerebral artery; PCOM, internal carotid-posterior communicating artery junction; SR, size ratio.

**Figure 2:**
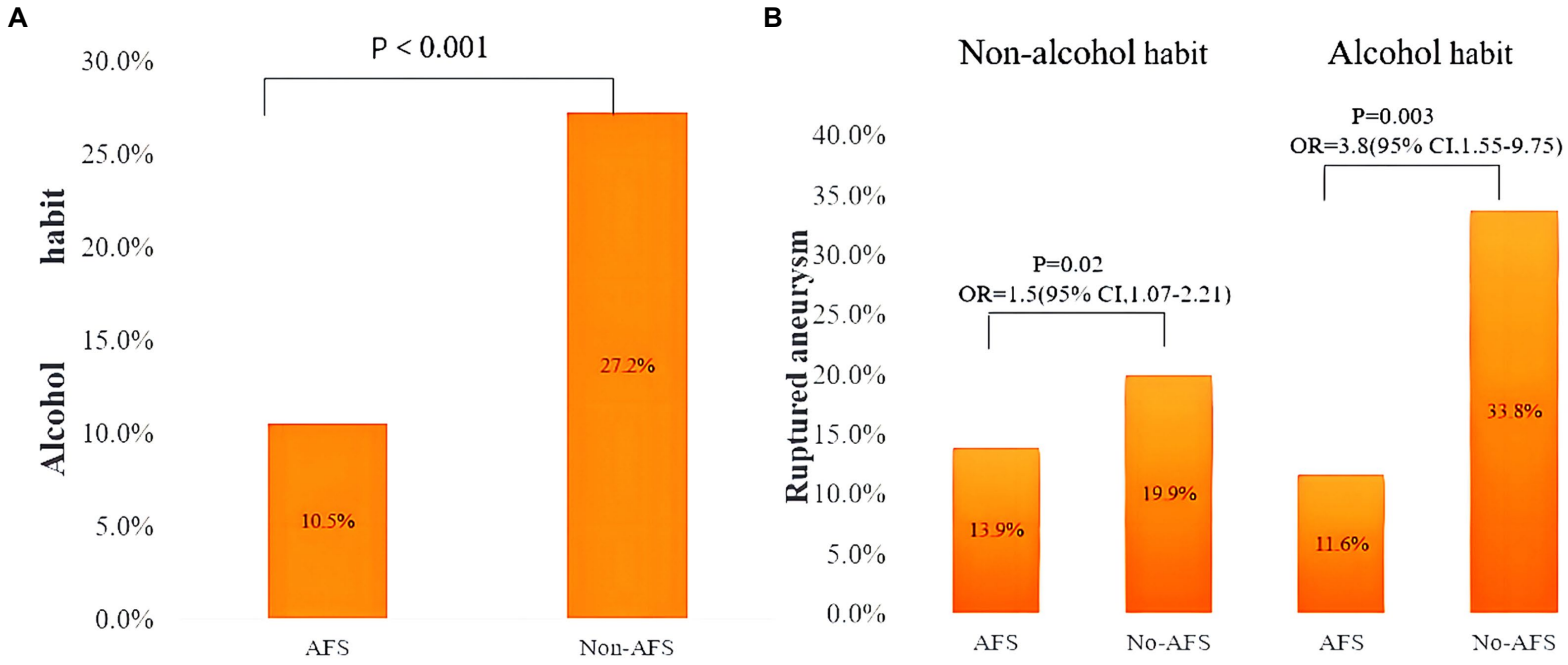
**(A)** Prevalence of habitual alcohol drinking in patients with and without alcohol flushing syndrome. **(B)** Prevalence of ruptured aneurysm with patients stratified according to both habitual alcohol drinking and presence of alcohol flushing syndrome. OR, odds ratio; CI, confidence interval; AFS, alcohol flushing syndrome.

Table 4 shows patient characteristics overall and according to AFS status. The incidence of aneurysm rupture was significantly higher in patients without AFS (*p* < 0.001). Meanwhile, there was a significantly difference between the AFS and non-AFS group in habitual alcohol consumption (10.5 vs. 27.2%, *p* < 0.001; Figure 2A).

**Table 4:** Clinical characteristics of patients grouped according to presence of alcohol flushing syndrome.

Characteristic	Total patients (*n* = 1,170)	No-AFS (*n* = 760)	AFS (*n* = 410)	*p* Value
Age				0.035
< 50, *n* (%)	327 (28.0)	197 (25.9)	130 (31.7)	
≥ 50, *n* (%)	843 (72.1)	563 (74.1)	280 (68.3)	
Sex				0.183
Male, *n* (%)	469 (40.1)	294 (38.7)	175 (42.7)	
Female, *n* (%)	701 (59.9)	466 (61.3)	235 (57.3)	
History of diabetes mellitus, *n* (%)	150 (12.8)	96 (12.6)	54 (13.2)	0.792
History of hypertension, *n* (%)	660 (56.4)	438 (57.6)	222 (54.2)	0.251
History of hyperlipidemia, *n* (%)	401 (34.3)	251 (33.0)	150 (36.6)	0.221
History of heart disease, *n* (%)	135 (11.5)	84 (11.1)	51 (12.4)	0.479
History of ischemic stroke, *n* (%)	154 (13.2)	97 (12.8)	57 (13.9)	0.582
Smoking，*n* (%)	339 (29.0)	217 (28.6)	122 (29.8)	0.665
Alcohol habit, *n* (%)	250 (21.4)	207 (27.2)	43 (10.5)	<0.001
Ruptured aneurysms, *n* (%)	236 (20.2)	180 (23.7)	56 (13.7)	<0.001
Multiplicity, *n* (%)	125 (10.7)	85 (11.2)	40 (9.8)	0.451

AFS, alcohol flushing syndrome.

### Univariate and multivariate analyses

In the univariate analyses (Table 5), female sex (OR 1.63; 95% CI, 1.22–2.17), history of ischemic stroke (OR 0.42; 95% CI, 0.25–0.72), smoking (OR 1.68; 95% CI, 1.25–2.25), habitual alcohol drinking (OR 1.97; 95% CI, 1.43–2.70), AFS (OR 0.49; 95% CI, 0.34–0.72), aneurysm size ≥ 7 mm (OR 3.39; 95% CI, 2.53–4.53), bifurcation location (OR 6.63; 95% CI, 4.46–9.85), posterior circulation location (OR 1.93; 95% CI, 1.18–2.79), irregular shape (OR 4.04; 95% CI, 2.97–5.49), aneurysm AR (OR 1.60; 95% CI, 1.31–1.97), aneurysm SR (OR 1.51; 95% CI, 1.38–1.64), and aneurysm HWR (OR 2.67; 95% CI, 1.76–4.06) were significantly associated with IAR.

**Table 5:** Results of univariate and multivariate regression analyses.

Characteristic	Univariate analysis		Multivariate analysis		*p*-Value	OR (95% CI)	*p*-Value	OR (95% CI)
Female	0.001	1.63 (1.22–2.17)	0.16	1.35 (0.89–2.06)
Age	0.07	0.99 (0.97–1.01)		
History of diabetes mellitus	0.78	0.94 (0.61–1.45)		
History of hypertension	0.193	1.21 (0.91–1.62)		
History of hyperlipidemia	0.095	0.77 (0.56–1.05)		
History of heart disease	0.687	1.09 (0.71–1.70)		
Smoking	0.001	1.68 (1.25–2.25)	0.094	1.49 (0.94–2.36)
Alcohol habit	< 0.001	1.97 (1.43–2.70)	0.876	1.04 (0.65–1.66)
Alcohol flushing syndrome	< 0.001	0.49 (0.34–0.72)	< 0.001	0.50 (0.35–0.71)
History of ischemic stroke	0.002	0.42 (0.25–0.72)	< 0.001	0.38 (0.22–0.65)
Posterior circulation aneurysms	0.006	1.93 (1.18–2.79)	0.463	1.20 (0.73–1.98)
Bifurcation	< 0.001	6.63 (4.46–9.85)	< 0.001	4.90 (3.6–8.32)
Irregular shape	< 0.001	4.04 (2.97–5.49)	< 0.001	2.87 (2.05–4.01)
≥7 mm	< 0.001	3.39 (2.53–4.53)	< 0.001	2.17 (1.46–3.24)
Aspect ratio	< 0.001	1.60 (1.31–1.97)	0.164	0.80 (0.58–1.10)
Size ratio	< 0.001	1.51 (1.38–1.64)	< 0.001	1.25 (1.12–1.41)
Height/width ratio	< 0.001	2.67 (1.76–4.06)	0.073	1.54 (0.96–2.46)

OR, odds ratio; CI, confidence interval.

In the multivariate analysis (Table 5), AFS (OR 0.50, 95% CI 0.35–0.71), history of ischemic stroke (OR 0.38; 95% CI, 0.22–0.65), aneurysm size ≥ 7 mm (OR 2.17; 95% CI, 1.46–3.24), bifurcation location (OR 4.9; 95% CI, 3.6–8.32), irregular shape (OR 2.87; 95% CI, 2.05–4.01), and aneurysm SR (OR 1.25, 95% CI 0.96–2.46) were independent predictors of aneurysmal rupture.

Because of the significant interaction between AFS and habitual alcohol drinking, we performed an analysis with patients stratified according to both categories. Among patients who did not habitually drink alcohol, the prevalence of ruptured aneurysm in the AFS and no AFS subgroups was 13.9 and 19.9%, respectively, and the odds of IAR were significantly higher in those without AFS (OR 1.54; 95% CI, 1.07–2.21; *p* = 0.02). Among habitual drinkers, the prevalence of IAR in the AFS and no AFS subgroups was 11.6 and 33.8%, respectively, and the odds of IAR were also significantly higher in those without AFS (OR 3.88; 95% CI, 1.55–9.75; *p* < 0.001; Figure 2B). Univariate analysis (Table 6) showed that hypertension (OR 1.54; 95% CI, 1.06–2.22), history of ischemic stroke (OR 0.37; 95% CI, 0.18–0.77), and AFS (OR 0.63; 95% CI, 0.44–0.92) were significantly associated with IAR in patients without a drinking habit; in the multivariate analysis, AFS (OR 0.69; 95% CI, 0.49–0.96) was independently associated with IAR. In habitual alcohol drinkers, multivariate analysis showed that AFS (OR 0.11; 95% CI, 0.03–0.45) was also independently associated with IAR.

**Table 6:** Univariate and multivariate regression analyses for rupture of intracranial aneurysm in patients with and without a drinking habit.

Characteristic	Non-habitual drinkers				Habitual drinkers				Univariate analysis		Multivariate analysis		Univariate analysis		Multivariate analysis		*p*-Value	OR (95% CI)	*p*-Value	OR (95% CI)	*p*-Value	OR (95% CI)	*p*-Value	OR (95% CI)
Female	0.132	0.70 (0.44–1.11)			0.183	0.98 (0.95–1.01)		
Age	0.334	0.99 (0.97–1.01)			0.514	1.52 (0.43–5.36)		
History of diabetes mellitus	0.987	1.00 (0.57–1.75)			0.364	0.71 (0.34–1.48)		
History of hypertension	0.022	1.54 (1.06–2.22)	0.128	1.50 (0.93–1.83)	0.328	0.76 (0.43–1.33)		
History of hyperlipidemia	0.120	0.74 (0.50–1.08)			0.221	0.70 (0.40–1.24)		
History of heart disease	0.726	0.90 (0.48–1.66)			0.207	1.57 (0.78–3.16)		
Smoking	0.680	1.12 (0.65–1.95)			0.551	1.28 (0.57–2.85)		
AFS	0.016	0.63 (0.44–0.92)	0.042	0.69 (0.49–0.96)	0.002	0.10 (0.02–0.41)	0.002	0.11 (0.03–0.45)
History of ischemic stroke	0.007	0.37 (0.18–0.77)	0.015	0.42 (0.21–0.85)	0.050	0.43 (0.18–1.00)	0.191	0.53 (0.20–1.37)

AFS, alcohol flushing syndrome; OR, odds ratio; CI, confidence interval.

### Discussion

Our study is the first to identify AFS as a possible new clinical marker for assessing the risk of intracranial aneurysm rupture. Conventional factors like history of ischemic stroke, bifurcation location, irregular shape, aneurysm size ≥ 7 mm, and aneurysm SR were also independently associated with aneurysmal rupture.

Facial flushing in patients with AFS occurs because of expansion of facial blood vessels and a temporary increase in facial blood flow after drinking alcohol (29). The underlying cause is accumulation of acetaldehyde, an alcohol metabolite (30). A subset of Asians, including the Chinese Han population, have a mutation in the ALDH2 gene that generates inactive ALDH2, an enzyme that scavenges and detoxifies acetaldehyde and other toxic aldehydes (12). These Asians have a higher prevalence of facial flushing than those without the mutation (31, 32). Epidemiological surveys have shown that ALDH2 gene polymorphisms are strongly linked to an increased incidence of stroke and cardiovascular risk factors (33). Several studies have identified the ALDH2*2 allele as an independent risk factor for ischemic stroke and cerebral infarction in the Chinese population (22, 34). Additionally, studies have indicated that the ALDH2*1 allele appears to be a significant risk factor for ischemic stroke (35), multiple lacunar infarction (36), and stroke (37, 38) in Asian populations.

AFS is a reliable proxy marker for ALDH2 genetic variants in East Asian populations and can be used to identify subjects with ALDH2 deficiency (16–18). In our study, we identified patients with AFS using an alcohol flushing questionnaire and found that AFS was negatively associated with IAR in a population of Chinese Han patients with intracranial aneurysms. One possible explanation for this is that the prevalence of habitual drinkers without AFS is higher than that of patients with AFS. Patients with AFS may experience facial flushing, headache, palpitations, and nausea after drinking alcohol. These adverse physiological reactions to alcohol consumption can reduce the patient’s dependence on alcohol and reduce alcohol intake (12, 15, 39). We compared the clinical characteristics of patients with and without AFS and showed that prevalence of habitual drinking and aneurysm rupture were higher in patients without AFS. Notably, previous studies, including the work by Can et al. (40), have found a significant correlation between alcohol use and the risk of IAR. ALDH2 polymorphisms might be linked to aneurysmal rupture because of their association with alcohol drinking behavior. Wang et al. (37) showed that the ALDH2*2 allele, which is phenotypically expressed as AFS, has a protective effect against stroke in Han Chinese individuals with a history of heavy alcohol consumption. The study also found that excessive alcohol intake can worsen ischemic brain injury by suppressing ALDH2 gene activity (39). However, AFS was independently associated with IAR in our multivariate logistic regression model, which adjusted for all other rupture risk factors, including habitual drinking. Analysis with patients grouped according to drinking habits showed that AFS was independently associated with IAR after adjusting for other confounding factors, regardless of drinking habits (Table 6). Therefore, the effect of AFS on aneurysmal rupture may be independent of drinking behavior. One possible explanation is that ALDH2 may have other effects on blood vessels in addition to its effects on alcohol metabolism. ALDH2 is expressed in multiple organs, such as the liver, kidney, and myocardium (41, 42), and has the potential to influence hormonal and lipid metabolism systems due to its polymorphisms. Several studies have shown that ALDH2 is associated with nitric oxide production in the vascular endothelium (43, 44), which may lead to endothelial apoptosis and oxidative stress (36). These effects may promote structural remodeling and fragility in aneurysm walls that eventually result in rupture (45, 46). In addition, Yang et al. (11) found that ALDH2 deficiency reduces the risk of aortic aneurysm and dissection in mice and humans *via* microRNA-mediated phenotypic switching of aortic vascular wall cells. However, the relevance of this mechanism in intracranial aneurysms has not been investigated.

In our study, 86.9% of RIAs were located at a bifurcation. In the multivariate model, bifurcation location was independently associated with increased odds of rupture (OR, 4.9). At an arterial bifurcation, the arterial wall is constantly in a weakened state because of high hemodynamic pressure and high blood flow, which explains the higher risk of rupture at these locations (47). In addition, aneurysm size ≥ 7 mm was also independently associated with rupture (OR, 2.17), which is consistent with previous reports (8, 48). However, Aoki (49) reported that SR, not aneurysm size, predicts rupture in UIAs. The increased risk of rupture with increasing SR is attributed to the presence of more dangerous hemodynamic features in aneurysms with higher SR (50). Our study also found that both SR and irregular shape are independently associated with rupture.

Interestingly, history of ischemic stroke was associated with lower odds of aneurysmal rupture. Statins and antiplatelet agents are commonly used for prevention of stroke in patients with hyperlipidemia and those with a previous ischemic stroke. Several recent studies have emphasized the potential protective effect of these agents for aneurysmal rupture (51–56). Potential mechanisms include anti-inflammatory effects, stimulation of extracellular matrix production, and chemotactic migration of mesenchymal progenitor cells to stabilize aneurysm walls (57, 58).

To the best of our knowledge, the relationship between AFS and intracranial aneurysm rupture has not been investigated in other populations. However, there is considerable subjectivity in AFS data obtained *via* questionnaires alone. Several studies have shown the presence of the alcohol dehydrogenase 2*2 allele in East Asians, which encodes a hyperactive form of alcohol dehydrogenase 2 that presents with AFS because acetaldehyde accumulates faster than ALDH2 can metabolize it (59); however, this polymorphism is found in a very low percentage of the population (17). Although we found an association between AFS and intracranial aneurysm rupture, single nucleotide sequencing would be required to determine the responsible gene and polymorphism.

This study has several other limitations. First, it was retrospective and conducted in a single center, so bias may have been introduced. Moreover, our data did not include amount of alcohol consumed, which almost certainly influences the association between AFS and aneurysmal rupture. Second, the alcohol flushing questionnaire used did not evaluate alcohol-related symptoms other than flushing, nor did it assess AFS severity. This lack of discrimination could potentially overestimate the accuracy of identifying ALDH2 gene mutations. Questionnaires may be more sensitive in detecting alcohol-related symptoms such as sleepiness, nausea, headache, and throbbing, according to several studies (60, 61). Finally, the characteristics of an aneurysm, such as AR, size, and SR, may be subject to change following rupture, which could have introduced bias in previous research.

## Conclusion

AFS may be a novel clinical marker to assess the risk of intracranial aneurysm rupture. Subgroup analysis and multivariate regression analysis revealed that the association between AFS and IAR exists independently of alcohol consumption. This implies that there may be a potential pathophysiological link between ADLH2 gene polymorphisms and the development and rupture of intracranial aneurysms. Further single nucleotide polymorphism testing and molecular biology studies are warranted.

## Data availability statement

The original contributions presented in the study are included in the article/supplementary material, further inquiries can be directed to the corresponding authors.

## Ethics statement

The studies involving human participants were reviewed and approved by Ethics Committee of Beijing Tiantan Hospital. The patients/participants provided their written informed consent to participate in this study.

## Author contributions

YL and ML: conception and design. XC, SG, and DD: data collection and patient follow-up. SG, LD, LZ, DW, JJ, HG, and PL: analysis and interpretation of data. XC and SG: drafting the article. XC, SG, DD, LD, LZ, DW, JJ, HG, PL, ML, and YL: critical revision. YL: approval of the final version on behalf of all authors. All authors contributed to the article and approved the submitted version.

## Funding

The National Natural Science Foundation of China (grant nos. 82271319 and 82171289) and Natural Science Foundation of Hebei Province of China (grant no. F2020202053) supported this study.

## Conflict of interest

The authors declare that the research was conducted in the absence of any commercial or financial relationships that could be construed as a potential conflict of interest.

The reviewer BJ declared a shared affiliation with the author(s) to the handling editor at the time of review.

## Publisher’s note

All claims expressed in this article are solely those of the authors and do not necessarily represent those of their affiliated organizations, or those of the publisher, the editors and the reviewers. Any product that may be evaluated in this article, or claim that may be made by its manufacturer, is not guaranteed or endorsed by the publisher.
